# Anti-Inflammatory Activities of Marine Algae in Neurodegenerative Diseases

**DOI:** 10.3390/ijms20123061

**Published:** 2019-06-22

**Authors:** Maria Cristina Barbalace, Marco Malaguti, Laura Giusti, Antonio Lucacchini, Silvana Hrelia, Cristina Angeloni

**Affiliations:** 1 Department for Life Quality Studies, Alma Mater Studiorum-University of Bologna, 40126 Bologna, Italy; maria.barbalace2@unibo.it (M.C.B.); marco.malaguti@unibo.it (M.M.); 2 School of Pharmacy, University of Camerino, 62032 Camerino, Italy; laura.giusti@unicam.it; 3 Department of Clinical and Experimental Medicine, University of Pisa, Pisa 56126, Italy; antonio.lucacchini@gmail.com

**Keywords:** neuroinflammation, neurodegeneration, algae, seaweeds, neurodegenerative diseases

## Abstract

Neuroinflammation is one of the main contributors to the onset and progression of neurodegenerative diseases such as Alzheimer’s and Parkinson’s diseases. Microglial and astrocyte activation is a brain defense mechanism to counteract harmful pathogens and damaged tissues, while their prolonged activation induces neuroinflammation that can trigger or exacerbate neurodegeneration. Unfortunately, to date there are no pharmacological therapies able to slow down or stop the progression of neurodegeneration. For this reason, research is turning to the identification of natural compounds with protective action against these diseases. Considering the important role of neuroinflammation in the onset and development of neurodegenerative pathologies, natural compounds with anti-inflammatory activity could be good candidates for developing effective therapeutic strategies. Marine organisms represent a huge source of natural compounds, and among them, algae are appreciated sources of important bioactive components such as antioxidants, proteins, vitamins, minerals, soluble dietary fibers, polyunsaturated fatty acids, polysaccharides, sterols, carotenoids, tocopherols, terpenes, phycobilins, phycocolloids, and phycocyanins. Recently, numerous anti-inflammatory compounds have been isolated from marine algae with potential protective efficacy against neuroinflammation. This review highlights the key inflammatory processes involved in neurodegeneration and the potential of specific compounds from marine algae to counteract neuroinflammation in the CNS.

## 1. Introduction

Neurodegeneration refers to a progressive and permanent loss of neurons in specified regions of the brain and spinal cord. It is the pathological condition that characterizes many neurodegenerative diseases, including Alzheimer’s disease (AD), Parkinson’s disease (PD), multiple sclerosis (MS), Huntington’s disease (HD), amyotrophic lateral sclerosis (ALS) [1], and traumatic brain injury (TBI) [2]. The main cellular and molecular events that trigger neurodegeneration are oxidative stress, abnormal protein deposition, damaged mitochondrial function, induction of apoptosis, impairment of proteostasis, and neuroinflammation [3]. Since the first identification of the main neurodegenerative disorders, research on the molecular mechanisms underlying these pathologies has focused on major anatomical changes such as neuronal loss and protein aggregation [4]. In recent years, more and more studies have highlighted the key role of the immune system in the initiation and progression of neurodegeneration [5,6] due to changes in cytokine signaling, immune cell proliferation and migration, altered phagocytosis, and reactive gliosis as common features of neurodegeneration [4]. Neuroinflammation, or, more specifically, the activation of the neuroimmune cells microglia and astrocytes into proinflammatory states, is an effective endogenous defense that protects the central nervous system (CNS) against microorganisms and injuries. It is usually a positive mechanism that aims to eliminate threats and restore homeostasis [7]. However, prolonged neuroinflammatory events can lead to a series of events that conclude with progressive neuronal damage that characterizes many neurodegenerative disorders [8]. The glial cells, microglia and astrocytes, have a pro- and anti-inflammatory role and are involved in different functions under physiological and disease conditions, such as phagocytosis, steroid release, free radical reduction, and cellular repair [9]. Glial cells exert a proinflammatory action through the production of cytokines and reactive oxygen species (ROS) that lead to synaptic dysfunction, loss of synapses, and neuronal death resulting in CNS injury. Until now, most research has been focused on microglial cells as key actors of neuroinflammation in neurodegeneration, but recently new scientific evidence has shown the important contribution of astrocytes to the inflammation that characterizes neurodegenerative diseases [10,11,12]. Unfortunately, to date there are no pharmacological therapies able to slow down or stop the progression of these devastating pathologies. For this reason, research is turning to the identification of natural compounds with protective action against these diseases. Considering the important role of neuroinflammation in the onset and development of neurodegenerative pathologies, natural compounds with anti-inflammatory activity could be good candidates to develop effective therapeutic strategies. Marine organisms represent a huge source of natural compounds, some of which have different structural characteristics from those of terrestrial origin. Marine-derived natural compounds could produce different pharmacological effects, like anti-diabetic [13], anti-inflammatory [14], antioxidant [15], anticancer [16], and anti-obesity [17] activities, and open the way for the development of new drugs [18]. Of note, seven marine-derived natural compounds have been approved for clinical use [19].

Among marine organisms, algae are one of the most valuable resources of the sea. Epidemiological studies comparing Japanese and Western diets show an association between algae consumption and a lower incidence of chronic degenerative diseases [20]. Algae are appreciated sources of important bioactive components such as antioxidants, proteins, vitamins, minerals, soluble dietary fibers, polyunsaturated fatty acids, polysaccharides, sterols, carotenoids, tocopherols, terpenes, phycobilins, phycocolloids, and phycocyanins [20]. Recently, Fernando et al. [21] summarized the latest knowledge about the potential anti-inflammatory activity of marine algae derivatives, evidencing their potential protective efficacy against neuroinflammation too. In particular, marine algae have been shown to counteract neuroinflammation by acting at different cellular levels: inhibiting pro-inflammatory enzymes such as COX-2 and iNOS [22], modulating MAPK pathways [23], and NK-kB activation [24], among others. Currently there are no clinical trials on the effects of marine algae against neuroinflammation but, given their important biological activities, as demonstrated by in vitro and animal studies, we believe that they will be carried out in the near future. Moreover, as anti-inflammatory drugs can trigger complications and important side effects [25,26], identifying novel anti-inflammatory agents from marine algae could be a valid solution to overcome this problem. In fact, anti-inflammatory natural compounds have been demonstrated to be safe thanks to their long use in folk medicine [27].

This review highlights the key inflammatory processes involved in neurodegeneration and the potential of marine algae and specific compounds from marine algae to counteract neuroinflammation in the CNS. The most recent and relevant results on the promising anti-inflammatory activities of marine algae related to neuroprotection have been selected.

## 2. Methods

A PubMed search was conducted. The combinations of terms that we used for this search were “marine algae and neuroinflammation,” “marine algae and clinical studies,” “marine algae and inflammation,” “marine algae and toxicity,” and “marine algae.” We also combined the terms marine algae and neuroinflammation with fucosterol, phlorotannins, astaxanthin, polysaccharides, glycoprotein, chlorophyll, lutein, zeaxanthin, violaxanthin, neoxanthin, or β-carotene. No restrictions were placed on the date of the articles or the language of publication. Studies with a clearly described methodology were included.

## 3. Molecular Mechanisms of Neuroinflammation

Neuroinflammation is a defense process aimed to protect both the brain and the spinal cord from tissue damage or pathogen invasion [8]. Generally, inflammatory processes involve numerous cellular types and mediators with the aim of separating, via the formation of a glial scar, damaged tissue from healthy tissue [28]. When an insult occurs at brain level, the immune response is mediated through cross-talk between the CNS and the periphery. In fact, due to inflammation, blood‒brain barrier (BBB) permeability is increased and leucocytes can infiltrate into the CNS [9].

At the brain level, microglia, astrocytes, and oligodendrocytes constitute the neuroglial cells [29]. Microglia have been demonstrated to be derived from primitive macrophages [30] and are now considered the resident immune system of the brain [31]. In non-activated conditions, microglia contribute to brain homeostasis [32] by modulating neuronal survival and maintenance thanks to the ability to release neurotrophic factors such as basic fibroblast growth factor and nerve growth factor (NGF) [33]. Acting as immune cells, microglial cells are also responsible for the phagocytosis of cell debris and contribute to the apoptosis of defective cells [34,35]. More recently, astrocytes, which are known to be involved in CNS homeostasis by sustaining synapse plasticity, have also been demonstrated to participate in protective signaling pathways such as those modulated by glycoprotein gp130, which is crucial for glial cells’ survival [36], and by the transforming growth factor beta (TGFβ), whose signaling has been shown to exert immunosuppressive effects and to inhibit nuclear factor κB (NF-κB) nuclear translocation [37].

Beside their neuroprotective properties, the microglia supervise the brain environment by modulating the immune functions in response to tissue damage, degeneration, and pathogen infections [38]. Their activation can be triggered by different stimuli such as lipopolysaccharide (LPS), a well-known toll-like receptor (TLRs) ligand [39], and they represent the first line of defense against infections [40]. Microglia activation results in both morphological and biochemical changes: cells lose their shape and begin to secrete inflammatory biomarkers such as cytokines, eicosanoids, nitric oxide, and ROS [41,42].

Even though neuroinflammation does not usually trigger neurodegenerative diseases, it is directly involved in neuronal dysfunctions and contributes to neuronal death and to neurodegenerative disease progression [43]. In fact, diseases such as PD, AD, ALS, and MS, as well as ischemia and TBI, are associated with chronic inflammation and long-lasting microglia activation [44]. Such chronic inflammatory states result in an abnormal increased cytokine levels [45], the production of neurotoxic mediators, and oxidative stress that triggers a pro-inflammatory cycle [46] and amplifies degenerative processes such as abnormal protein deposition, mitochondrial dysfunction, and BBB permeability impairment [44,47,48].

Chronic inflammation in neurodegenerative diseases is sustained by TLRs activation at the glial level [49]. Among TLRs, TLR4 is the most expressed in microglia [50]; its activation has been demonstrated to be responsible for chronic inflammation in AD, where Aβ-oligomers interact with TLR4 and increase its expression [51,52], and in PD, where TLR4 protein expression is also increased in both in vitro and in vivo model systems [53]. Moreover, TLR4 has been found to be responsible for inflammation in spinal cord injury and stroke [54]. TLR4 activation triggers two different downstream proinflammatory signaling pathways, leading to cytokine expression. Among these pathways, the phosphoinositide 3-kinase/protein kinase B (PI3K/Akt) pathway, mammalian target of rapamycin (mTOR) activation, and mitogen-activated protein kinases cascades (MAPKs) are the main ones involved and lead to NF-κB activation [7,55,56]. Once activated, PI3K triggers Akt phosphorylation, which in turn activates mTOR. The mTOR pathway plays a pivotal role in the regulation of NF-κB and inflammation [57]. NF-κB signaling is considered particularly important in every neuroinflammation-related disease. After initial TLR4 activation, the sequence of events that leads to the translocation of NF-κB to the nucleus includes the activation of the protein IκB kinase, phosphorylation of the IκB inhibitory protein, and the consequent release of active NF-κB [58]. As a dimer, NF-κB translocates to the nucleus, where it activates the transcription of its target genes such as inducible nitric oxide synthase (iNOS), cyclooxygenase (COX2), tumor necrosis factor alpha (TNF-α), interleukin (IL)-6, and IL-1β by binding to p65-responsive element [56]. During neuroinflammation, NF-κB signaling is also stimulated in astrocytes [59], where its translocation to the nucleus and the subsequent cytokines expression is triggered by IL-17-receptor [60] and lactosyl ceramide, a lipid mediator produced by astrocytes [61]. Astrocytes’ contribution to neuroinflammation and neurotoxicity has, thus, been demonstrated in models of different neurodegenerative diseases such as brain injury [62] and spinal cord and nerve injury [63,64], where NF-κB inactivation resulted in positive outcomes.

MAPKs are proteins involved in the regulation of multiple cellular functions. In particular, they are involved in the regulation of apoptosis, cell differentiation, and proliferation.

In activated microglia, increased signaling of p38 MAPK and c-Jun N-terminal kinases (JNK) has been described [65]. These MAPKs induce, through the transcription factor activating protein-1 (AP-1), the transcription of proinflammatory genes such as COX2, TNF-α, and IL-6. The involvement of p38 and JNK signaling in the LPS-activated MG6 microglial cell line has recently been confirmed, showing that LPS treatment strongly induces phospho-p38/p38 and phospho-JNK/JNK ratio, the AP-1 translocation to the nucleus, iNOS protein expression, and NO production [65].

PI3K/Akt and MAPK are not the only pathways involved in neuroinflammation; the Janus Kinase/Signal Transducers and Activators of Transcription (JAKs/STATs) signaling pathway represents a further pathway able to trigger inflammation in the CNS [66]. Several cytokines trigger this pathway by binding their specific receptors and promoting JAK kinase activity, both in microglia and astrocytes. Once activated, JAK phosphorylates STAT, which dimerizes and translocates to the nucleus, where it promotes the expression of cytokine-responsive genes. At least four JAK and seven STAT proteins have been identified [67]. Specific combinations of JAKs and STATs are involved in the response to different cytokines, allowing each cytokine to transduce its own message [66]. JAKs/STATs are involved in the inflammatory response occurring in most neurodegenerative diseases. In MS, endoplasmic reticulum stress induces astrocyte activation through JAK1/STAT3 signaling [68]. IL-6 and IFN-γ, two major activators of JAKs/STATs signaling, are elevated in PD [69]; moreover, in primary microglial cell culture it has been demonstrated that the inhibition of JAK 1/2 prevents the release of NO, TNF-α, and IL-1β induced by α-synuclein treatment [70,71].

Besides classical inflammatory pathways, non-classical pathways, such as the Hippo pathway, have been related to neuroinflammation and in particular to astrocyte activation [72]. In its typical sequence of events, the Hippo pathway involves numerous kinases such as Mst 1/2, Sav1, and Last 1/2. Last 1/2 phosphorylates and thus inactivates by proteasomal degradation or cytoplasmic retention, two transcription factors: YAP and TAZ. When dephosphorylated YAP and TAZ migrate to the nucleus, where they promote the expression of downstream genes [73]. YAP has been found to be highly expressed in astrocytes and its deletion induced astrocytic activation in both cell cultures and in vivo studies [72]. In astrocytes, IFNβ induced YAP activation, which, in turn, promoted the expression of the suppressor of cytokine signaling 3 (SOCS3), a negative regulator of JAK-STAT. In fact, YAP(-/-) astrocytes showed hyperactivation of the JAK-STAT pathway and astrocyte activation [72].

Neuroinflammation represents a crucial aspect of neurodegenerative disease progression. Targeting neuroinflammatory pathways seems to be a promising strategy to counteract neurodegenerative diseases. As different pathways are involved in the onset of neuroinflammation, compounds with different molecular targets are the best candidates to fight this condition. On these bases, beside drug development, the study of natural bioactive compounds, thanks to their varied and complex structures, can help with the identification of effective anti-inflammatory agents.

## 4. Marine Algae

Algae are photosynthetic eukaryotic organisms that present a complex and controversial taxonomy. More than 20,000 species of algae have been identified, and on the basis of their size they are divided in macroalgae (seaweeds) and microalgae. Macroalgae are multicellular marine plants, while microalgae are small unicellular or simple multicellular species [74]. Marine macroalgae can be classified into three classes according to their pigments: Brown (Phaeophyta) Green (Chlorophyta), and Red (Rhodophyta). The pigments responsible for the algae’s color are: fucoxanthin (Phaephyta); chlorophyll a, b, lutein, zeaxanthin violaxanthin neoxanthin, and β-carotene (Chlorophyta); phycobilliproteins and lutein, zeaxanthin, and β-carotene (Rhodophyta). The classification of microalgae is extremely complex considering the thousands of species present even in small areas of water.

Microalgae are classified into groups based on different characteristics: pigment composition, morphological variations (rounded, oval, cylindrical, and fusiform cells), the presence of thorns, cilia, flagella etc. In addition, they can be classified based on their sizes: picoplankton (0.2–2 μm), nanoplankton (2–20 μm), and microplankton (20–200 μm). Recently, Corrêa et al., at the 16th IEEE International Conference on Machine Learning and Applications in 2017, proposed a deep learning technique to solve the problem by using as input low-resolution images [75].

Marine algae are composed of various substances: carbohydrates, lipids, proteins, amino acids, vitamins, minerals, and secondary metabolites such as phytosterols and polyphenols [76]. The chemical composition of macroalgae is considerably different between species and dependent on the season (sunlight), habitat (salinity, depth in the sea), and environmental conditions.

### 4.1. Carbohydrates

Among the various components, carbohydrates are the most abundant constituents of marine algae. Moreover, polysaccharides are usually the major component of red, green, and brown algae [77,78], and monosaccharides and oligosaccharides are also present. The storage polysaccharide is laminarin in brown algae and floridean in starch (more branched than amylopectin) in green and red algae. Algae cell walls are characterized by the presence of uncommon polysaccharides that can be sulfated, acetylated, etc. Marine algae carbohydrates are promising compounds in various fields, such as food, pharmaceutical, and biomedical. Noteworthy therapeutic applications are due to their antiviral, antibacterial, and antitumoral activities, antioxidant, antilipidemic, and antiglycemic properties, and anti-inflammatory and immunomodulatory characteristics. In particular, alginate-derived oligosaccharides inhibit neuroinflammation [79]. Laminarin (a polysaccharide composed of (1,3)-β-d-glucan with β(1,6) branching), particularly abundant in *Laminaria* species, has been demonstrated to possess antibacterial and chemopreventive activities, together with prebiotic activity [80], important in modulating gut microbiota, which in turn can regulate neuroinflammation [81]. Algae polysaccharides have been also utilized in the cosmeceutical industries due to their chemical and physical properties exhibiting potential benefits for skin [82].

Table 1 shows the different carbohydrates of brown, green, and red macroalgae. The oligosaccharides derived from polysaccharides are also important. They are produced by chemical or enzymatic hydrolysis and present numerous activities such as antioxidant, anti-inflammatory, and anti-melanogenic [83,84,85,86,87]. Microalgae also produce polysaccharides, and release in particular sulfated polysaccharides (carrageenan, ulvan, and fucoidan) [88,89,90]. Polysaccharides found in the cell wall vary among microalgae genera and species. Microalgae present an advantage with respect to macroalgae because they are easy to grow and culture and do not depend on the climate or season.

To date the ability of algae-derived polysaccharides to counteract neuroinflammation has not yet been fully explored.

### 4.2. Lipids

Algae contain different types of lipid phospholipids, non-polar glycerolipids, glycolipids, betaine lipids, and some unusual lipids, e.g., sulfolipid (sulfoquinovosyldiacylglycerol) sterols [91].

Marine macroalgae have a low lipid content but the proportion of long-chained polyunsaturated fatty acids (PUFA) is relatively high. In macroalgae, PUFAs are represented by omega-3 and omega-6 fatty acids. The content of PUFAs is generally higher in those living in cold water. Eicosapentaenoic acid (EPA) is the principal fatty acid. PUFAs have health benefits: they regulate blood clotting and blood pressure and develop functions of the brain and nervous systems [92,93]. They also decrease the risk of many chronic diseases such as arthritis, diabetes, and obesity [94,95], and regulate the signaling of microglia, mostly in the context of neuroinflammation and behavior [93].

*Sterols*. Among macroalgae, cholesterol is the most representative sterol in all the red algae; fucosterol, which has anti-inflammatory activity, is the chief sterol in brown algae [96], and in green algae the dominant sterol is isofucosterol clionasterol. Microalgae are characterized by the presence of unusual dihydroxysterols, pavlovols, crinosterols, and stigmasterols. It has been proposed that sterols, due to their ability to cross the blood‒brain barrier, can prevent neuroinflammation [97,98], but there are few reports of the neuroprotective activities of algae-derived phytosterols.

### 4.3. Proteins and Amino Acids

Macroalgae and microalgae have been used as a source of human nutrition for thousands of years by some indigenous populations. This is due to their significant protein content, which is even greater than some ground plant sources. Algae proteins are rich in aspartic and glutamic acid, the latter contributing to the typical taste (umami). Green macroalgae, and especially red macroalgae, have a higher protein content than brown macroalgae. Macroalgae also contain a number of bioactive amino acids and peptides (e.g., taurine, carnosine, and glutathione and mycosporine-like) [99] that have been demonstrated to exert antioxidant and antiapoptotic effects in the rat brain [100]. Lectins are a group of glycoproteins isolated from algae [101] that present several properties including anti-inflammatory [102,103] antibiotic, cytotoxic, mitogenic, antinociceptive, and anti-viral due to their ability to bind to specific glycan structures [104]. Marine algae, with their high protein content, are now considered a precious source of bioactive peptides, obtained after enzymatic digestion, with considerable health potential. These biopeptides have been demonstrated to exhibit antioxidant, anticancer, antihypertensive, antiatherosclerotic, and immunomodulatory activities [105]. In the future it is desirable that research address the potential neuroprotective role of these biopeptides, elucidating their mechanism of action.

### 4.4. Phenols

Phenolic compounds are a class of chemical compounds characterized by hydroxyl groups directly attached to aromatic hydrocarbon rings. The simplest is composed of one aromatic ring and is called phenol. Phenolic compounds can be single phenols or polyphenols, depending on the number of phenol units in the molecule.

Phenols are largely represented in all the organisms belonging to the Plant kingdom; however, the phenols present in marine algae are different to those produced by terrestrial plants [104].

The best known polyphenols in marine algae are phloroglucinols and phlorotannins. Phlorotannins can be classified into subclasses: eckols, fuhalols, fucophlorethols, phlorethols, fucols, and ishofuhalols.

The largest proportion of phenolic compounds is in green and red algae (bromophenols, phenolic acids, and flavonoids). Phlorotannins are found only in marine brown algae [106,107].

Phenols and polyphenols from marine algae have attracted much attention for their anticancer, antioxidant, antimicrobial, and anti-inflammatory activities [108]. To, date several mechanisms behind microglial activation have been reported (see Section 3), and research is moving towards the discovery of alternative anti-inflammatory compounds from natural renewable sources that could potentially counteract neuroinflammation and, therefore, neuronal injury in neurodegenerative diseases, characterized by complex and deeply related phenomena. Marine algae rich in phenols are good candidates for potential application in the nutraceutical sector.

### 4.5. Isoprenoids

Carotenoids and terpenoids are two important classes of isoprenoids belonging to the marine algae. Carotenoids contains eight isoprene units, while terpenoids contain five isoprene units. 

The carotenoids that consist of only hydrocarbons are carotenes, while those with oxo, hydroxyl, or epoxy groups are called xanthophylls. The most diffuse carotenoids in marine algae are: β-carotene, fucoxanthin, astaxanthin, canthaxanthin, and lutein. Fucostantin is mostly present in brown algae and in planktonic microalgae, while β-carotene is predominant in green microalgae [109,110].

The potential health-promoting effects of these carotenoids are: antioxidant activity, anti-inflammatory effects, anticancer activity anti-obese effect, antidiabetic activity, hepatoprotective effect, antiangiogenic effect, and cerebrovascular protective effect [111,112,113]. In particular, fucoxanthin has been demonstrated to decrease inflammation and oxidative damage [114] and astaxanthin has been demonstrated to decrease the expression of IL-6 in activated microglial cells [115], all factors implicated in the pathogenesis of neurodegenerative diseases.

Brown macroalgae are considered one of the principal source of biologically and ecologically relevant terpenoids, mainly diterpenes and meroditerpenes [116]. In *Sargassum*, meroterpenoids prevail, in particular sargachromenol, which presents anti-inflammatory and neuroprotective effects. Also, green algae are a source of terpenes, in particular the genus *Caulerpa*, which is represented by about 60 species living in tropical and subtropical waters that biosynthesize acyclic and monocyclic sesqui- and diterpenes [117] with neuroprotective activities.

## 5. Marine Algae and Neuroinflammation

As previously mentioned, activated microglia are a critical modulator of the neuroinflammation process, triggering a self-feeding loop with the neighboring astrocytes through the release of pro-inflammatory cytokines, including TNF-α and IL-1β [118]. In this context, a persistent and unrestrained neuroinflammatory loop harms neuronal cells and can promote neurodegenerative diseases [119]. Recent years have been characterized by a huge boost in nutritional research to discover natural compounds with anti-inflammatory properties and potential neuroprotective capacity. Marine algae have been part of a healthy diet in East Asia for centuries and represent a rich reservoir of structurally different bioactive compounds with great potential for pharmaceutical applications. Increasingly, reports have shown the anti-inflammatory action of marine algae [120], as well as of their major components such as phlorotannins and pigments [121,122,123].

The methanol extract of *Ulva conglobata,* a green alga consumed as a marine vegetable, has been demonstrated to possess anti-inflammatory potential [22]. In particular, the extract was tested in hippocampal neuronal HT22 cells and microglial BV2 cells. In HT22 cells, 40 and 50 µg/mL *Ulva conglobata* extract was able to significantly restore cellular viability compared to glutamate-treated cells. Moreover, *Ulva conglobata* extract effectively suppressed IFN-γ-induced microglial activation, and 50 µg/mL inhibited NO release and reduced the expression of iNOS and COX-2 enzymes. Kim et al. [124] found that the hexane fraction of brown seaweed *Myagropsis myagroides* ethanolic extract exhibits the highest anti-inflammatory activity among different solvent fractions. In LPS-stimulated BV-2 cells, 25 µg/mL *Myagropsis myagroides* extract had the potential to revert the induction of pro-inflammatory mediators such as NO, PGE_2_, and the cytokines IL-6 and TNF-α through the prevention of NF-κB nuclear translocation and MAPKs phosphorylation. Surprisingly, they did not identify the active compound responsible for these effects. Meanwhile, another report from the same authors suggested that the anti-inflammatory activity of *Myagropsis myagroides* ethanolic extract in LPS-stimulated BV-2 cells could be completely ascribed to the presence of sargachromenol [125]. A study assessed the anti-neuroinflammatory capacity of three extracts obtained from Malaysian seaweed: *Padina australis, Sargassum polycystum*, and *Caulerpa racemosa* [126]. All the extracts reduced the elevation of inflammatory mediators like NO, TNF-α, IL-6, and IL-1β, with the brown seaweeds (*Padina*, *Sargassum)* showing stronger inhibitory activity compared to the green seaweed (*Caulerpa*).

The so-called “cholinergic hypothesis” suggests a correlation between memory impairment in AD and the reduction of neurotransmitter acetylcholine [127]. The preservation of acetylcholine levels could be useful in view of a multitarget therapy. Fucosterol, a sterol mainly found in brown algae including *Padina australis,* was isolated to investigate its cholinesterase and inflammatory inhibitory properties [128]. It was observed that fucosterol inhibits acetylcholinesterase (AChE) and butyrylcholinesterase (BChE), both responsible for acetylcholine hydrolysis, and significantly prevents the production of pro-inflammatory mediators in LPS-induced C8-B4 microglial cells and in Aβ-induced BV-2 microglial cells.

*Ecklonia cava,* an edible brown alga used for the production of food ingredients, animal feed, and fertilizers, has been shown to possess anti-inflammatory activity [129,130].

Three of the major phlorotannins that can be found in *Ecklonia cava* eckol, dieckol, and 8,8’-bieckol, were investigated for their protective effects against Aβ_25-35_-induced neuroinflammatory damage in PC12 cells [130]. The results indicated that all phlorotannins tested possess antioxidant and protective effects against Aβ damage, while dieckol has the strongest ability to combat apoptosis and Ca^2+^ overload and more effectively inhibits the increase of inflammatory markers and the protein levels of p65, the best studied NF-κB subunit. Therefore, the neuroprotective property of dieckol with a diphenyl ether linkage was greater than that of 8,8’-bieckol with a biaryl linkage, although these two compounds are both dimers of eckol.

These data were further confirmed by Jung et al. [129], who isolated dieckol from *Ecklonia cava* extract, reporting its potential as an anti-inflammatory agent by reducing the release and stimulation of pro-inflammatory cytokines and enzymes together with an intracellular scavenging activity. Also, a component from *Ecklonia stolonifera*, phlorofucofuroeckol B, was identified as a potent suppressor of inflammation, inhibiting IκB-α/NF-κB and Akt/ERK/JNK pathways [23]. A study conducted by Kim et al. [131] demonstrated, for the first time, that floridoside, a natural glycerol galactoside from the red alga *Laurencia undulata*, possesses the potential to counteract the neuronal damage induced by neuroinflammation in vitro, preventing ROS and NO overload due to iNOS and COX-2 overexpression. Among algae pigments, fucoxanthin is one of the main carotenoids found in brown algae [132]. In an Aβ_42_ -induced microglial activation model, fucoxanthin significantly reduced the rates of inflammatory and oxidative damage, protecting DNA from oxidation and attenuating the increasing of inflammatory enzymes [114]. Astaxanthin, a red carotenoid pigment, occurs naturally in plants and marine seaweeds, but also in shellfish and crustaceans [133]. It has been shown to possess a variety of pharmacological effects, including anti-inflammatory and antioxidative activity [133,134,135,136].

Increasing evidence correlates a neuronal inflammation status with the development of depression [137,138]. In a rat model of LPS-induced depressive-like behaviors, 80 mg/kg astaxanthin had an antidepressant-like effect due to the restoration of LPS-induced alterations of brain inflammatory markers (i.e., IL-1β, IL-6, and TNF-α), as well as iNOS, nNOS, and COX-2 expression via the modulation of NF-κB activation [24].

In addition, Zhang et al. [139] found that astaxanthin administration could alleviate early brain injury via suppressing the inflammation damage induced by subarachnoid hemorrhage. In particular, 75 mg/kg astaxanthin significantly reduced the elevated cortical levels of inflammatory mediators, together with the degree of neutrophil infiltration.

A food supplement approved by the U.S. Food and Drug Administration (FDA), named Aquamin, is a natural multi-mineral derived from the marine red seaweed *Lithothamnion corallioides.* Aquamin was evaluated for its anti-neuroinflammatory potential, and in cortical glial-enriched cells was able to suppress the release of LPS-induced TNF-α and IL-1β. Recently, several authors suggested that anti-inflammatory and antioxidative agents could prevent the deposition of Aβ and the subsequent brain damage [140,141]. Indeed, in the promoter of neuronal beta-secretase 1 (BACE1), the enzyme involved in Aβ buildup, NF-κB DNA consensus sequences are present [142]. So, it could be beneficial in treating AD to reduce microglia-mediated neuroinflammation and increase microglia scavenger activity for toxic Aβ aggregates [143]. The ethanol extract of *Nannochloropsis oceanica* demonstrated anti-inflammatory, antioxidative, and anti-amyloidogenesis activities in a mouse model of LPS-induced AD [141]. The authors recently found that the main component of *Nannochloropsis oceanica* is eicosapentaenoic acid (EPA), suggesting that it could be responsible for the neuroprotective effects. The depolymerization of the polysaccharide alginate, found in many marine brown algae, produces alginate-derived oligosaccharide with various biological activities depending on the degradation method used [79]. The alginate-derived oligosaccharide produced by enzymatic depolymerization showed anti-inflammatory activity by repressing the LPS and Aβ-induced production of inflammatory cytokines and mediators in microglial cells. These effects have been associated with the inactivation of the TLR4/NF-κB axis [79]. Interestingly, the interaction between this oligosaccharide and TLR4 promotes the uptake of toxic Aβ aggregates. Regarding the possibility of alginate-derived oligosaccharide crossing the BBB, the authors declared an average molecular weight of 1500 Da and previous works demonstrated that oligosaccharides produced by enzymatic depolymerization are able to pass through the BBB easily [25,144]. Differently, Bi et al. [13] synthesized a seleno-polysaccharide from alginate-derived polymannuronate. Using in vitro/in vivo models of microglia and astrocyte activation, the pre-treatment with seleno-polymannuronate reduced the overgeneration of proinflammatory mediators, including NO, PGE_2_, TNF-α, IL-6, and IL-1β as well as iNOS and COX-2, by suppressing the MAPK/NF-κB signaling pathway. Cui et al. [145] assessed whether fucoidan, a class of fucose-enriched sulfated polysaccharides isolated from *Laminaria japonica,* protects dopaminergic neurons from inflammation-mediated damage in a PD inflammatory rat model induced by an intranigral injection of LPS. Fucoidan was able to improve behavioral deficits in mice by protecting them from the loss of dopaminergic neurons. Other important anti-AD and anti-inflammatory effects have been manifested by the glycoproteins purified from brown alga *Undaria pinnatifida* [146]. *Undaria pinnatifida* displayed dose-responsive inhibition for AChE and BChE with an IC_50_ of 63.56 and 99.03 µg/mL, respectively, and has been shown to inhibit BACE1, acting on the neurotransmitter acetylcholine and on the formation and accumulation of Aβ aggregates. Moreover, *Undaria pinnatifida* promotes cell survival and neurite extension, preventing inflammation status.

Epidemiological studies demonstrate a negative correlation between the use of non-steroidal anti-inflammatory drugs (NSAIDs) and the incidence of inflammation in the nervous system, which in turn participates in the development of neurodegenerative diseases [120]. The NSAIDs’ mechanism of action involves the inhibition of the inflammatory mediator release. Marine algae can control the inflammatory process in microglia, suggesting their potential role as neuroprotective agents. Moreover, the signaling pathways involved in the neuroprotective activity of algae are multiple. The complexity of neurodegenerative diseases makes them difficult to counteract with single-target molecules. In this context, marine algae, with their pleiotropic effects, have a great potential for application as anti-neuroinflammatory agents. However, further studies are needed, along with clinical trials to confirm marine algae’s anti-neuroinflammatory activity.

## 6. Conclusions

The wide range of biological and bioactive molecules found in marine algae represents a challenge for researchers involved in the study of neuroinflammation/neurodegeneration processes. Marine algae extracts and many marine algae constituents belonging to different chemical classes have been demonstrated to exert preventive/protective effects against neuro-inflammation (Table 2). In particular, they have been demonstrated to be effective in reducing inflammatory mediators like NO, TNF-α, IL-6, and IL-1β, in downregulating inflammatory enzymes like iNOS and COX-2, and in modulating the signaling pathways that lead to NF-κB activation. Moreover, most of the compounds isolated from marine algae have also shown antioxidant activity. Oxidative stress represents a hallmark of neuroinflammation and its counteraction could be a successful strategy in the prevention of neurodegeneration. ROS production is strictly related to neuro-inflammation, and marine algae compounds with both antioxidant and anti-inflammatory activities are good candidates to counteract neurodegeneration thanks to their pleiotropic activity. A better knowledge of these molecules should be associated with an implementation in the extraction and purification procedures in order to obtain marine algae extracts with standardized concentrations to be applied in in vitro studies. In fact, the choice of an appropriate extraction method can deeply influence the presence and concentration of the bioactive compounds. Moreover, the ability of marine algae constituents to cross the blood‒brain barrier has not been investigated, which calls into question the possibility of developing them as neuroprotective agents. Also, studies on potential adverse effects are lacking. Although still in their infancy, studies on the anti-neuroinflammatory effects of marine algae compounds should be corroborated by clinical trials. Currently there is a paucity of information reported in the literature, which only contains studies on in vitro or animal models. Human studies could strengthen the choice of marine algae products as potential nutraceutical compounds for the prevention of neuro-inflammation.

## Figures and Tables

**Table 1:** Carbohydrates in marine algae.

Carbohydrates	Brown Macroalgae	Red Macroalgae	Green Macroalgae
monosaccharides	glucose, galactose, xylose, fucose, uronic acid, glucuronic acid mannuronic acid, guluronic acid	glucose, galactose, mannose	glucose, mannose, xylose, rhamnose, glucuronic acid, uronic acid
polysaccharides	laminarin alginate, fucoidan (sulphated), cellulose, mannitol	carrageenans (sulfated), agar (sulfated), floridean starch, cellulose, lignin, funoran	ulvan (sulfated), mannan, galactans (sulfated), xylans, floridean starch, cellulose, lignin

**Table 2:** Studies showing anti-neuroinflammatory activities of marine algae.

Marine Algae Extract/Bioactive Compound	Treatment Conc.	Experimental Model	Key Findings
*Ulva conglobata* methanol extract	10‒50 µg/mL	mouse hippocampal HT-22 cells; mouse microglial BV-2 cells	Restoration of cellular viability in HT-22 cells; downregulation of COX-2 and iNOS in BV-2 cells [22]
Exane fraction of *Myagropsis myagroides* ethanolic extract	5‒25 µg/mL	mouse microglial BV-2 cells	Decreased release of inflammatory cytokines, inactivation of NF-κB and reduced mRNA and protein levels of iNOS and COX-2 [124]
*Myagropsis myagroides* ethanolic extract	5–25 µg/mL	mouse microglial BV-2 cells	Reduction in NO, PGE_2_, IL-6, IL-1β and TNF-α release; inhibition of ERKs-JNKs/NF-κB axis [125]
*Padina australis*, *Sargassum polycystum* and *Caulerpa racemosa* extracts	0.05–0.4 mg/mL	mouse microglial C8-B4 cells	Decreased release of pro-inflammatory mediators (NO, PGE_2_, IL-6, IL-1β and TNF-α) [126]
Fucosterol from *Padina australis*	0.004–192 µM	mouse microglial C8-B4 and BV-2 cells	Inhibition of AChE and BChE; reduction in release of NO, PGE_2_, IL-6, IL-1β and TNF-α in LPS-stimulated C8-B4 cells; prevented production of NO, IL-6 and TNF-α in Aβ_42_-stimulated BV-2 cells [128]
Eckol, dieckol and 8,8’-bieckol from *Ecklonia cava*	1–50 µM	rat neuronal PC12 cells	Antioxidant activity; anti-apoptotic effects; decrease in key inflammatory proteins (COX-2, iNOS, IL-1β and TNF-α) [130]
Dieckol from *Ecklonia cava*	50–300 µg/mL	mouse microglial BV-2 cells	Inhibition of LPS-induced iNOS and COX-2 protein and mRNA expression; suppression of p-38/ NF-κB pathway; ROS scavenging activity [129]
Phlorofucofuroeckol B from *Ecklonia stolonifera*	10–40 µM	mouse microglial BV-2 cells	Inhibition of IκB-α/NF-κB and Akt/ERK/JNK pathways [23]
Floridoside from *Laurencia undulata*	1–50 µM	mouse microglial BV-2 cells	Inhibition of LPS-induced NO and ROS production; downregulation of COX-2 and iNOS mRNA and protein levels by reducing p38 and ERK phosphorylation [131]
Fucoxanthin	5–50 µM	mouse microglial BV-2 cells	Attenuation of Aβ_42_-induced cytokines release (NO, PGE_2_, IL-6, IL-1β and TNF-α) and enzymes upregulation (COX-2, iNOS) by suppressing MAPKs phosphorylation; protection from H_2_O_2_-induced ROS release and DNA damage by recovering antioxidant enzymes [114]
Astaxanthin	20–80 mg/Kg	male ICR mice	Reversed LPS-induced depressive-like behaviors; attenuation of cytokines level (IL-6, IL-1β and TNF-α) and antagonization of iNOS, nNOS and COX-2 expression in the hippocampus and prefrontal cortex [24]
Astaxanthin	75 mg/Kg	male Sprague‒Dawley rats	Amelioration in cerebral edema, blood‒brain barrier disruption, neurological dysfunction and neuronal degeneration after the induction of subarachnoid hemorrhage; downregulation of NF-κB activity, and intercellular adhesion molecule-1, IL-1β and TNF-α expression [139]
Aquamin^TM^	0.05–2 mg/mL	cortical glial-enriched cultures from Sprague‒Dawley rat pups	Attenuation of LPS-induced IL-1β and TNF-α secretion [147]
*Nannochloropsis oceanica* ethanol extract	50–100 mg/Kg	male ICR mice	Decrease of ROS and malondialdehyde levels; improvement of LPS-induced memory impairment; suppression of Aβ_42_ generation by downregulating APP and BACE1 expression [141]
Alginate-derived oligosaccharide	50–500 µg/mL	mouse microglial BV-2 cells	Inhibition of LPS/ Aβ_42_-induced NO and PGE_2_ production, COX-2 and iNOS expression, and cytokines secretion; attenuation of TLR4 and NF-κB overexpression; promotion of Aβ phagocytosis [79]
Seleno-polymannuronate	0.5 mg/mL, 0.8 mg/mL	primary microglia and astrocytes from BALB/c mouse pups; female BALB/c mice	In LPS-activated primary cells, attenuation of NF-κB and MAPK signaling with the reduction of NO, PGE_2_ production, downregulation of COX-2 and iNOS expression, and IL-6, IL-1β and TNF-α secretion; decrease of Iba1- and GFAP-positive cells in the brain of a mouse model of LPS-induced inflammation [13]
Fucoidan	7.5 mg/Kg, 15 mg/Kg; 31.25–125 µg/mL	male Sprague‒Dawley rats; primary microglia from neonatal Sprague–Dawley rats	Improvement of behavioral deficits and prevention of dopaminergic neuron loss; inhibition of ROS and TNF-α release [145]
Glycoprotein from *Undaria pinnatifida*	5–45 µg/mL	primary hippocampal cells from embryonal Sprague–Dawley rats	Inhibition of AChE, BChE and BACE1; promotion of cell survival and neurite extension [146]

## Abbreviations

AChE	Acetylcholinesterase
AD	Alzheimer’s disease
ALS	Amyotrophic lateral sclerosis
AP-1	Activating protein 1
A-	Amyloid beta
BACE1	Beta-secretase 1
BBB	Blood‒brain barrier
BChE	Butyrylcholinesterase
CNS	Central nervous system
COX-2	Cyclooxygenase-2
EPA	Eicosapentaenoic acid
HD	Huntington’s disease
IL	Interleukin
iNOS	Inducible nitric oxide synthase
JNK	c-Jun N-terminal kinases
LPS	Lipopolysaccharide
MAPKs	Mitogen-activated protein kinases cascade
MS	Multiple sclerosis
NF-κB	Nuclear factor κB
NGF	Nerve growth factor
nNOS	Neuronal nitric oxide synthase
NO	Nitric oxide
NSAIDs	Non-steroidal anti-inflammatory drugs
PD	Parkinson’s disease
PGE_2_	Prostaglandin E2
PUFA	Polyunsaturated fatty acids
TBI	Traumatic brain injury
TGFβ	Transforming growth factor beta
TLRs	Toll-like receptor
TNF-α	Tumor necrosis factor alpha
